# Food insecurity, body mass index, socio-economic status, and food intake in lactating and non-lactating mothers with children under two years

**DOI:** 10.1186/s40795-023-00718-9

**Published:** 2023-04-17

**Authors:** Milad Rajabzadeh-Dehkordi, Fatemeh Mohammadi-Nasrabadi, Mehran Nouri, Ali Ahmadi, Shiva Faghih

**Affiliations:** 1 Department of Community Nutrition, School of Nutrition and Food Sciences, Shiraz University of Medical Sciences, Shiraz, Iran; 2 Students’ Research Committee, Shiraz University of Medical Sciences, Shiraz, Iran; 3 Food and Nutrition Policy and Planning Research Department, Faculty of Nutrition Sciences and Food technology, National Nutrition & Food Technology Research Institute (NNFTRI), Shahid Beheshti University of Medical Sciences, Tehran, Iran; 4 Health Policy Research Center, Institute of Health, Shiraz University of Medical Sciences, Shiraz, Iran; 5 Modeling in Health Research Center, Shahrekord University of Medical Sciences, Shahrekord, Iran; 6 Nutrition Research Center, Shiraz University of Medical Sciences, Shiraz, Iran

**Keywords:** Food insecurities, Nutrients, Obesity, Chi-square test, Demographic facilities, Women

## Abstract

**Background:**

food insecurity (FI) is considered as an essential public health problem which may have detrimental effects on people’s health. The aim of present study was to evaluate FI, body mass index, quantity and quality of food intake in lactating and non-lactating mothers with children under two years.

**Methods:**

in this cross-sectional study 307 mothers (237 lactating and 70 non-lactating) were participated. Socio-economic and demographic information were gathered by questionnaires. FI of families was evaluated by the questionnaire of the United States Department of Agriculture (USDA) Household Food Security. For assessing quality and quantity of food intake of mothers, dietary diversity score (DDS), diet quality index-international (DQI-I) and nutrient adequacy ratio (NAR) were calculated. Weight and height of participants were measured and body mass index (BMI) was calculated. Finally, Chi-squared test, analysis of variance (ANOVA) and linear regression were used for statistical analysis.

**Results:**

in this study the rate of underweight, normal weight, overweight, and obesity in mothers was 0.3%, 39.2%, 42.3%, and 18.2%, respectively. Among the determinants of BMI, household food security status had the greatest effect (Beta=-1.584, P < 0.001) and mother age had the least effect (Beta = 0.101, P = 0.013). Mother’s occupational and educational status, having facilities, physiological status of mother, and house size had significant correlation with NAR. Mother’s occupational and educational status, and having facilities had significant relationship with DDS, too. Also, the significant correlation of Mother’s education, having facilities, and physiological status of mother with DQI-I were found.

**Conclusions:**

we found that Household food security status had the most effect on BMI of mothers. In this study, the best nutrient adequacy and dietary diversity were found in the obese group and the most diet quality was found in the normal weight group.

## Introduction

Food insecurity which is defined as the restricted access to food and failure to acquire enough food through socially admissible ways, has become a great public health concern in the world [1]. It is an intricate phenomenon and has different dimensions such as, cultural, social, psychological dimensions which can affect quality and quantity of life [2, 3].

Food insecurity (FI) has relation with deficiency of dietary diversity and the low intake of nutrients [4, 5].

In Asian region, FI was increased from 22.7 to 25.8% during the pandemic of coronavirus (COVID-19) [6]. FI can be influenced by different factors including age, family size, education and socio-economic status [7]. Nearly 800 million people in the world obtain inadequate food and their health is affected [6]. Based on a systematic review and meta-analysis, the prevalence of FI were 49% in Iranian families [8].

The nutritional condition of mothers and children is an appropriate index to survey the overall health of society and the status of food security in families [9]. Long term inadequate caloric intake can influence the amount and quality of breast milk and lead to infant malnutrition [10]. Mother and child under nutrition is a serious general health problem and was responsible for 45% of child deaths in 2011 [11]. Thus, it is necessary that the diet of mother have adequate calorie and nutrients [12].

A diverse diet is essential for achieving optimal nutrition adequacy and growth [13]. Few studies in Iran have examined the relationship between FI and anthropometric indices (such as weight and height) in mothers with children under two years. The hypothesis of present study was that, there was a relationship between FI and BMI. Therefore, in this study, we assessed the association between FI, body mass index, and food intake in lactating and non-lactating mothers with children under two years in Shahrekord, Iran.

## Methods

### Subjects

According to the previous similar article [14] and with considering the first type error equal to 0.05 and the power of 0.8 using NCSS (PASS) software, the sample size was estimated equal to 307 persons. Three hundreds and seven mothers (237 lactating and 70 non-lactating) participated in this cross-sectional study, who were selected from medical centers of Shahrekord (the center of Chaharmahal and Bakhtiari province, in the southwest of Iran). The mean age of mothers was 31.4 and the mean BMI of them was 26.5. Stratified random sampling was used to select the participants from April to June 2021 (Some detailed of the present study have been published previously [15]). In this study, the inclusion criteria was being the mother of a healthy child under two years’ old who visited Shahrekord health centers and the exclusion criteria was reporting daily energy intake was more than 4200 and less than 800 kcal. The purpose and method of the present study were explained to the participants and all of them completed a written consent form. All experiments were performed in accordance with relevant guidelines and regulations. The protocol of this study was approved by Ethics Committee Shiraz University of Medical Sciences (IR.SUMS.REC.1399.1022).

### Data collection

Data was collected by questionnaires through face-to-face interviews. Socio-economic and demographic information of participants including family size, house ownership type, number of employed members in the family, participants’ education and occupational status (employed/unemployed) were asked. To assess the economic status of the participants’ household, they were asked whether any of nine items including have: dishwasher, television, house, washing machine, car (Taxi or none Taxi), computer, refrigerator, hand woven carpet and microwave. This questionnaire was scored as follows: low: 3 items or less; moderate: 4–6 items and high: 7–9 items [14]. Physiological status of mother was measured and reported only based on breastfeeding or not breastfeeding. The United States Department of Agriculture (USDA) Household Food Security Survey Module [1] was used for assessing FI status of families in the last twelve months which was previously validated in Iran [16, 17]. It has 18 questions and categorized in this way: 0–2 scores: food secure; 3–7: mild food insecure; 8–12: moderate food insecure and 13–18: severe food insecure [1].

Dietary diversity score (DDS) was calculated by the method explained in Kant et al. study [18–20]. In this method, foods were divided into 5 groups including: grains, fruits, vegetables, dairy products, and meats. The grains group had seven components including: macaroni, refined bread, whole grain bread, rice, biscuits, corn flakes and refined flour. Fruits group was the summing up of fruits and fruit juices, citrus fruits and berries. Vegetables group consisted of tomatoes, potatoes, other starchy vegetables, green vegetables, yellow vegetables, legumes and other vegetables. Meats group included poultry, eggs, fish and red meat and dairy group consisted of cheese, yoghurt and milk. The score of each group was from 0 to 2, therefore the highest score was 10.

Diet quality index-international (DQI-I) was used for assessing diet quality. DQI-I includes four main components of the diet. First, variety of food (0–20 points) which has two components including between food groups (fish and shellfish, meat and products of meat, beans, eggs, milks and products of milk, grains, fruits, vegetables) and within protein sources group (fish and shellfish, meat and products of meat, beans, eggs, milks and products of milk). Second, adequacy (0–40 points) which assesses amounts of grains, fruits, vegetables, fibers, protein, calcium, vitamin C and ferric. The third, moderation (0–30 points) includes total fat, cholesterol, saturated fat, sodium and calorie-free foods. The fourth, overall balance (0–10 points) includes the ratio of fatty acids and the ratio of macronutrients. Finally, total DQI-I score was from 0 to 100 (0 means the minimum and 100 means the maximum diet quality) [21].

Nutrient adequacy was evaluated by nutrient adequacy ratio (NAR) method [22]. NAR was computed for vitamins A, C, D, E, B1, B2, B3, carbohydrate, protein, calcium, iron, magnesium, phosphorus, selenium, zinc, potassium and sodium.

### Anthropometric measurements

Weight and height of participants were measured based on standard protocols of WHO (with an accuracy of 0.1 kg for weight and 0.1 cm for height) [23], then body mass index (BMI) was calculated. Categorization of BMI was done as follows: less than 18.5: underweight; from 18.5 to 24.9: normal weight; from 25.0 to 29.9: overweight; and 30 Kg/m^2^ or more: obese [24].

### Statistical analysis

SPSS for windows software (version 25.0) was used for data analysis. P-value less than 0.05 was considered as significant. Analysis of variance (ANOVA) was used to examine for quantitative variables, and Chi-square test to examine the association between qualitative variables. Besides, linear regression was applied to find major determinants of participants’ BMI, nutrient adequacy ratio, diet diversity score, and diet quality index.

## Results

**Fig. 1:**
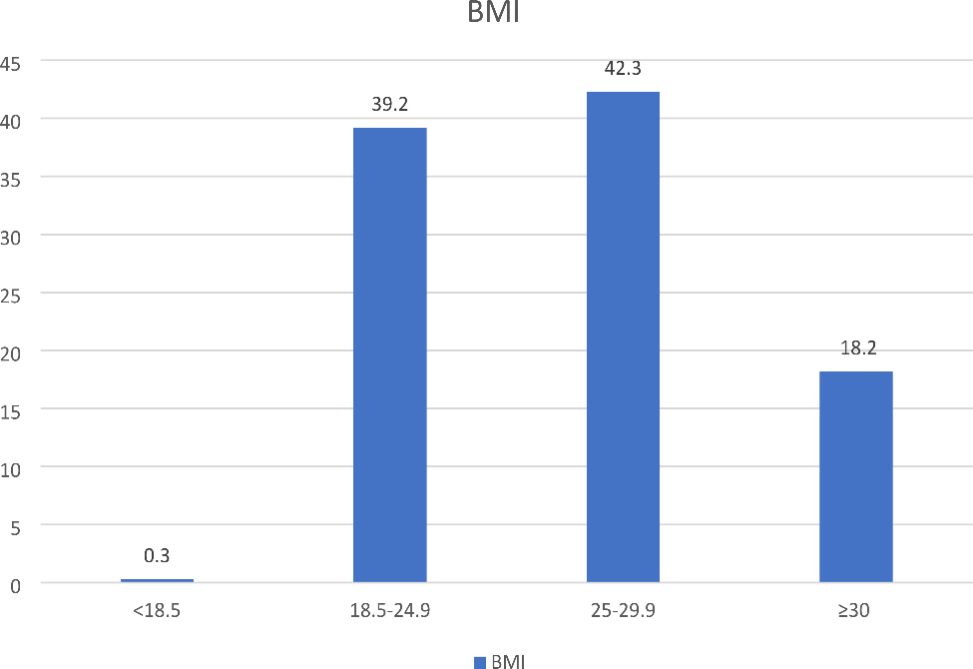
Relative frequency of underweight, normal weight, overweight, and obesity in mothers with children under two years based on their BMI

The frequency of underweight, normal weight, overweight, and obesity in mothers was 0.3%, 39.2%, 42.3%, and 18.2%, respectively (Fig. 1). The underweight group and normal weight group were mixed together and formed one group (BMI < 25) for advanced analysis.

As Table 1 shows, mother age was significantly different between BMI categorized groups (P = 0.020). The group of obese mothers had a higher average age and normal weight group had a lower average age. Household food security status which was divided into three groups was significantly different between three groups of BMI (P = 0.001). The highest percentage of food security was found in the obese group. Also, NAR (P = 0.003), DDS (P = 0.001), and DQI-I (P < 0.001) were significantly different between BMI groups. The most NAR and DDS were in the obese group and the most DQI-I was in the normal weight group.

**Table 1:** Socio-economic characteristics, household food security, and diet quality indices in mothers with children under two years based on their body mass index

Variables		Total (N = 307)	Mother BMI			P-value†
			BMI*<25	**25 ≤ BMI ≤ 29.9**	**BMI** ≥ 30	
**Mother age**	**Year** (mean ± SD)	31.4 ± 5.3	30.4 ± 5.0	32.1 ± 5.2	32.3 ± 5.6	0.02
**Mother’s occupational status**	**Housewife (%)** **Employed (%)**	78.8 21.2	79.3 20.7	75.4 24.6	85.7 14.3	0.62
**Mother ‘s education**	**Illiterate (%)** **Elementary (%)** **Middle school (%)** **Diploma (%)** **Above diploma (%)**	1.0 6.2 24.2 27.2 41.4	0.8 6.7 24.2 28.3 40.0	0.8 5.4 27.1 27.9 38.8	1.9 7.5 17.0 22.7 50.9	0.82
**Physiological status of mother**	**Breastfeeding (%)** **Not breastfeeding (%)**	77.2 22.8	76.9 23.1	76.2 23.8	80.4 19.6	0.81
**Father’s occupational status**	**Employed (%)** **Unemployed (%)**	98.4 1.6	96.6 3.4	99.2 0.8	100 0	0.15
**Father education**	**Illiterate (%)** **Elementary (%)** **Middle school (%)** **Diploma (%)** **Above diploma (%)**	1.0 4.3 19.7 38.5 36.5	2.6 3.4 20.5 34.2 39.3	0 4.7 18.8 39.8 36.7	0 5.6 20.4 44.4 29.6	0.50
**Family size**	**3–4 (%)** **5–7 (%)**	90.2 9.8	92.6 7.4	89.2 10.8	87.5 12.5	0.50
**House ownership type**	**Private house (%)** **Rental house (%)**	76.5 23.5	73.0 27.0	76.7 23.3	83.9 16.1	0.27
**Having the facility**	**Low (%)** **Moderate (%)** **High (%)**	11.7 51.5 36.8	17.4 46.3 36.3	9.2 56.2 34.6	5.4 51.8 42.8	0.09
**Household food security status**	**Food secure (%)** **Mild FI (%)** **Moderate &** **Severe FI (%)**	67.5 25.7 6.8	57.0 31.4 11.6	69.3 26.9 3.8	85.7 10.7 3.6	0.00
**NAR**	Mean ± SD	36.5 ± 7.7	34.7 ± 9.4	37.5 ± 6.0	38.1 ± 6.4	0.00
**DDS**	Mean ± SD	4.9 ± 1.8	4.5 ± 2.0	5.2 ± 1.6	5.3 ± 1.5	0.00
**DQI-I**	Mean ± SD	65.1 ± 4.4	66.1 ± 5.4	64.7 ± 3.7	63.2 ± 2.6	< 0.001
**Total (%)**		100	39.5	42.3	18.2	

*BMI: Body Mass Index, SD = Standard Deviation, FI = Food Insecurity, NAR: Nutrient Adequacy Ratio, DDS: Dietary Diversity Score, DQI-I: Diet Quality Index-International. P-value < 0.05 was considered significant

† ANOVA was used for quantitative variables and Chi-square test for qualitative variables

Economic status was assessed by a questionnaire and was classified according to appliances of life [14]: low: 3 or less than 3; moderate:4–6; high: 7–9

Among the determinants of BMI in linear regression model (the model was adjusted for Mother’s education, Economic status, Physiological status of mother, and DDS), household food security status had the greatest effect (Beta=-1.584, P < 0.001), Mother’s occupational status (Beta=-0.680, P = 0.030), DQI-I (Beta=-0.310, P < 0.001), NAR (Beta = 0.120, P = 0.005) and mother age (Beta = 0.100, P = 0.010) were ranked next (Table 2).

**Table 2:** Major determinants of body mass index in mothers with children under two years tested by the linear regression model

Predictors	Beta	P-value	95% CI for Beta
**Mother age (year)**	0.10	0.01	(0.02, 0.18)
**Mother’s occupational status**	-0.68	0.03	(-1.30, -0.06)
**Household food security status (USDA)**	-1.58	< 0.001	(-2.43, -0.72)
**NAR**	0.12	0.00	(0.03, 0.20)
**DQI-I**	-0.31	< 0.001	(-0.41, -0.21)

Dependent variable: BMI, P-value < 0.05 was considered significant

NAR: Nutrient Adequacy Ratio, DDS: Dietary Diversity Score, DQI-I: Diet Quality Index-International

The model was adjusted for Mother’s education, Economic status, Physiological status of mother, and Dietary Diversity Score (DDS).

According to Table 3, mother’s occupational status (Beta=-1.348, P = 0.023), mother’s education (Beta = 1.630, P = 0.002), having facilities (Beta = 0.963, P = 0.002), physiological status of mother (Beta = 3.441, P < 0.001), and house size (Beta=-0.036, P = 0.025) had significant association with NAR. Mother’s occupational status (Beta=-0.318, P = 0.025), Mother’s education (Beta = 0.402, P = 0.001), and having facilities (Beta = 0.319, P < 0.001) had significant correlation with DDS, too. Moreover, the significant correlation of Mother’s education (Beta = 1.420, P < 0.001), having facilities (Beta = 0.516, P = 0.006), and physiological status of mother (Beta = 0.794, P = 0.008) with DQI-I were found.

**Table 3:** Major determinants of nutrient adequacy ratio, diet diversity score, and diet quality index in mothers with children under two years by the linear regression model

Predictors	NAR			DDS			DQI-I		
	Beta	P-value	95% CI for Beta	Beta	P-value	95% CI for Beta	Beta	P-value	95% CI for Beta
**Mother age (year)**	0.02	0.76	(-0.13, 0.18)	0.01	0.39	(-0.02, 0.05)	-0.01	0.78	(-0.11, 0.08)
**Mother’s occupational status**	-1.34	0.02	(-2.50, -0.18)	-0.31	0.02	(-0.59, -0.04)	-0.47	0.18	(-1.17, 0.23)
**Mother’s education**	1.63	0.00	(0.62, 2.63)	0.40	0.00	(0.16, 0.64)	1.42	< 0.001	(0.80, 2.03)
**Economic status**	0.96	0.00	(0.35, 1.57)	0.31	< 0.001	(0.17, 0.46)	0.51	0.00	(0.14, 0.88)
**Physiological status of mother**	3.44	< 0.001	(2.47, 4.40)	0.14	0.23	(-0.09, 0.37)	0.79	0.00	(0.20, 1.38)
**Household food security status (USDA)**	-0.25	0.76	(-1.91, 1.40)	-0.21	0.29	(-0.61, 0.18)	0.56	0.26	(-0.43, 1.57)
**House size (m** ^**2**^ **)**	-0.03	0.02	(-0.06, -0.01)	-0.01	0.18	(-0.01, 0.01)	-0.00	0.69	(-0.02, 0.01)
**Family size**	0.37	0.54	(-0.85, 1.60)	-0.03	0.83	(-0.32, 0.26)	-0.00	0.98	(-0.75, 0.73)
**Father’s job**	-3.16	0.34	(-9.68, 3.35)	-0.75	0.34	(-2.31, 0.81)	-0.51	0.79	(-4.47, 3.44)
**Father education**	-0.54	0.32	(-1.63, 0.53)	-0.04	0.75	(-0.30, 0.22)	-0.47	0.15	(-1.13, 0.18)

Economic status was evaluated by a questionnaire and was classified according to appliances of life [14]: low: 3 or less than 3; moderate:4–6; high: 7–9

NAR: Nutrient Adequacy Ratio, DDS: Dietary Diversity Score, DQI-I: Diet Quality Index-International. P-value < 0.05 was considered significant

## Discussion

In this study, the prevalence of overweight in mothers was twice that of. However, in the study conducted by Mohammadi et al. [25] in Tehran-Iran, the number of overweight women is almost equal to the present study, but the difference is in the number of obese women, which can be due to different lifestyles and more sedentary life in big cities like Tehran.

We found that BMI was related to mother age, mother’s occupational status, household food security status, NAR and DQI-I. Among these factors, household food security status had the greatest positive impact. In one study in Lebanon, no significant relationship was found between FI in households and maternal overweight status and also this study concluded that FI in families was related with compromised dietary quality and diversity of mothers [26]. In contrast to the present study, a cross-sectional study in Mexico observed a positive association between severe FI in households and obesity in women [27].

One study in Iranian female in Tehran, observed that DQI-I had no relationship with BMI or waist circumference [28]. However, in a study in Guatemalan adults showed a positive correlation of DQI-I with BMI and waist circumference [29]. Another study in Mexico concluded that diet quality scores were not related to BMI of men and women and also said that dietary scores had inverse relationship with waist circumference just in men [30]. One study in Chinese adults who have type 2 diabetes showed that better quality of diet such as more dietary variety and reduced red meat intake was correlated with lower odds of obesity [31]. These differences can be due to various sample size, use different questionnaires and also various nutrition habits in different countries and even within a country, in big and small cities, the relationship may be different.

One study among mothers in Lebanon showed that, mothers in food insecure families were at high risk for obesity and dietary inadequacy [32]. A meta-analysis study showed that adults in food-insecure families, particularly women, are more at risk of obesity and also concluded that the risk of weight abnormality can increase with the exacerbation of FI level [33]. Food insecurity is adversely associated with dietary quality in US adults, particularly intakes of nutrient-rich vegetables, fruit, and dairy that promote good health [34].

The mechanism behind the association observed between food insecurity and BMI among women remains unclear and change in energy intake and change in physical activity do not exhaust the possible pathways through which FI could affect the women’s BMI in food insecure households [35]. Researchers have suggested a number of mechanisms, most having to do with low income mothers’ coping strategies, including managing limited resources of food and sacrificing their own nutrition in order to protect their children from hunger. It is believed that inadequate resources and putting children’s needs first, can create a chronic “feast or famine” situation, which appears to contribute to maternal obesity. Food deprivation can cause a preoccupation with food that has the potential to cause obesity, which results in overeating at the times during which they have access to adequate amounts of food. Women lacking adequate resources may be purchasing less expensive energy-dense foods, such as refined grains, sugar, and fat in order to stave off hunger, or avoiding fruits and vegetables because of their cost [25, 36].

In the present study, DDS had significant association with mother’s occupational status, mother’s education and having facilities. One study in Kermanshah-Iran, observed a significant association between DDS and having facilities, but DDS had no significant association with BMI or waist circumference and also, showed that DDS had no association with the level of education, which may be because the people participating in this study were employees of university of medical science and their level of nutritional knowledge was probably relatively good [37]. A study found that DDS was positively correlated with education of mother [38]. Education increases availability to information, growth income. Thus, an educated mother can have more ability to select food for her family. Differences in results may be due to geographical areas and having different culture which can affect consumption of foods.

Based on this study, DQI-I had relationship with mother’s education, having facilities and breastfeeding status. Similar to the present study, one study in Japan suggested that there is a positive relationship between diet quality and education in Japanese women [39].

The present study had some limitations including small sample size of study, not determining the cause-effect relationship in cross-sectional studies. The strength of this study is the assessing the effect of DDS, NAR and DQI-I on the BMI of mothers simultaneously.

## Conclusion

This study concluded that more than half of participants were suffered from some degrees of obesity. Household food security status had the most effect on BMI of mothers. More well-designed case-control or cohort studies are necessary to confirm the relationships between food insecurity, weight status, and diet quality in mothers and understand their effects on families in the future. According to the present study, the best nutrient adequacy and dietary diversity were observed in the obese group and the most diet quality was found in the normal weight group. So, planning interventions to improve BMI and food security status of mothers is very necessary.

## Data Availability

The datasets used and/or analyzed during the current study are available from the corresponding author on reasonable request.

## List of Abbreviations

FI: Food Insecurity
USDA: United States Department of Agriculture
DDS: Dietary Diversity Score
NAR: Nutrient Adequacy Ratio
DQI-I: Diet Quality Index-International
BMI: Body Mass Index
ANOVA: Analysis of Variance
SD: Standard Deviation

## References

[1] Bickel G, Nord M, Price C, Hamilton W, Cook J. Guide to measuring household food security, revised 2000. US Department of Agriculture, Food and Nutrition Service. 2000:52.

[2] Casey PH, Simpson PM, Gossett JM, Bogle ML, Champagne CM, Connell C (2006). The association of child and household food insecurity with childhood overweight status. Pediatrics.

[3] Lang T, Heasman M (2015). Food wars: the global battle for mouths.

[4] Mello JA, Gans KM, Risica PM, Kirtania U, Strolla LO, Fournier L (2010). How is food insecurity associated with dietary behaviors? An analysis with low-income, ethnically diverse participants in a nutrition intervention study. J Am Diet Assoc.

[5] Mohamadpour M, Sharif ZM, Keysami MA (2012). Food insecurity, health and nutritional status among sample of palm-plantation households in Malaysia. J Health Popul Nutr.

[6] Organization WH. The state of Food Security and Nutrition in the World 2021: transforming food systems for food security, improved nutrition and affordable healthy diets for all. Food & Agriculture Org.; 2021.

[7] Payab M, Dorosty A, Eshraghian M, Siassi F, Karimi T (2012). Association of food insecurity with some of socioeconomic and nutritional factors in mothers with primary school child in Rey city. Iran J Nutr Sci Food Technol.

[8] Behzadifar M, Behzadifar M, Abdi S, Arab Salmani M, Ghoreishinia G, Falahi E, et al. Prevalence of Food Insecurity in Iran: a systematic review and Meta-analysis. Prevalence of Food Insecurity in Iran; 2016. 27041526

[9] Alemayehu M, Argaw A, Mariam AG (2015). Factors associated with malnutrition among lactating women in subsistence farming households from Dedo and Seqa-Chekorsa districts, Jimma zone, 2014. Developing Ctry Stud.

[10] Kong X. Influencing factors from lactating mother without diseases on breast milk quality and quantity.International Journal of Pediatrics. 2016:445–8.

[11] Black RE, Victora CG, Walker SP, Bhutta ZA, Christian P, De Onis M (2013). Maternal and child undernutrition and overweight in low-income and middle-income countries. The lancet.

[12] Torheim LE, Arimond M. Diet quality, micronutrient intakes and economic vulnerability of women. Diet Quality: An Evidence-Based Approach, Volume 1. 2013:105 – 15.

[13] Vakili M, Abedi P, Sharifi M, Hosseini M (2013). Dietary diversity and its related factors among adolescents: a survey in Ahvaz-Iran. Global J health Sci.

[14] Safarpour M, Dorosty Motlagh A, Hosseini SM, Ranjbar Noshari F, Safarpour M, Daneshi Maskooni M (2014). Prevalence and outcomes of food insecurity and its relationship with some socioeconomic factors. Knowl Health.

[15] Rajabzadeh-Dehkordi M, Mohammadi-Nasrabadi F, Nouri M, Ahmadi A, Faghih S. Determinants and consequences of food insecurity in families having children under the age of 2 years.Nutrition and Health. 2022:02601060221135923. 10.1177/02601060221135923 36412042

[16] Ramesh T. The Prevalence of food insecurity and some associated factors among Shirazian households in 2009 [dissertation] Tehran: Shahid Beheshti University. MC; 2009.

[17] Rafiei M, Nord M, Sadeghizadeh A, Entezari MH (2009). Assessing the internal validity of a household survey-based food security measure adapted for use in Iran. Nutr J.

[18] Arthur Schatzkin M (1991). Dietary diversity in the US population, NHANES II, 1976–1980. J Am Diet Assoc.

[19] Kennedy G, Ballard T, Dop MC. Guidelines for measuring household and individual dietary diversity. Food and Agriculture Organization of the United Nations; 2011.

[20] Shirani M, Saneei P, Nouri M, Maracy M, Abbasi H, Askari G (2020). Associations of major dietary patterns and dietary diversity score with semen parameters: a cross-sectional study in iranian infertile men. Int J fertility Steril.

[21] Kim S, Haines PS, Siega-Riz AM, Popkin BM (2003). The Diet Quality Index-International (DQI-I) provides an effective tool for cross-national comparison of diet quality as illustrated by China and the United States. J Nutr.

[22] Hammond K, Mahan L, Intake. Analysis of the diet. Krause’s food and nutrition care process Missouri: Elsevier Saunders. 2012:129 – 43.

[23] De Onis M, Onyango AW, Van den Broeck J, Chumlea WC, Martorell R (2004). Measurement and standardization protocols for anthropometry used in the construction of a new international growth reference. FoodNutr Bull.

[24] Kuczmarski RJ, Carroll MD, Flegal KM, Troiano RP (1997). Varying body mass index cutoff points to describe overweight prevalence among US adults: NHANES III (1988 to 1994). Obes Res.

[25] Mohammadi F, Omidvar N, Harrison GG, Ghazi-Tabatabaei M, Abdollahi M, Houshiar-Rad A (2013). Is household food insecurity associated with overweight/obesity in women?. Iran J public health.

[26] Jomaa LH, Naja FA, Kharroubi SA, Diab-El-Harake MH, Hwalla NC (2020). Food insecurity is associated with compromised dietary intake and quality among lebanese mothers: findings from a national cross-sectional study. Public Health Nutr.

[27] Ponce-Alcala RE, Luna JLR-G, Shamah-Levy T, Melgar-Quiñonez H (2021). The association between household food insecurity and obesity in Mexico: a cross-sectional study of ENSANUT MC 2016. Public Health Nutr.

[28] Zamani B, Daneshzad E, Mofrad MD, Namazi N, Larijani B, Bellissimo N (2021). Dietary quality index and cardiometabolic risk factors among adult women. Iran J Public Health.

[29] Gregory CO, McCullough ML, Ramirez-Zea M, Stein AD (2008). Diet scores and cardio-metabolic risk factors among guatemalan young adults. Br J Nutr.

[30] López-Olmedo N, Popkin BM, Mendez MA, Taillie LS (2019). The association of overall diet quality with BMI and waist circumference by education level in mexican men and women. Public Health Nutr.

[31] Cheung LT, Chan RS, Ko GT, Lau ES, Chow FC, Kong AP (2018). Diet quality is inversely associated with obesity in chinese adults with type 2 diabetes. Nutr J.

[32] Jomaa L, Naja F, Cheaib R, Hwalla N (2017). Household food insecurity is associated with a higher burden of obesity and risk of dietary inadequacies among mothers in Beirut, Lebanon. BMC Public Health.

[33] Moradi S, Mirzababaei A, Dadfarma A, Rezaei S, Mohammadi H, Jannat B (2019). Food insecurity and adult weight abnormality risk: a systematic review and meta-analysis. Eur J Nutr.

[34] Hanson KL, Connor LM (2014). Food insecurity and dietary quality in US adults and children: a systematic review. Am J Clin Nutr.

[35] Nettle D, Bateson M. Food-insecure women eat a less diverse diet in a more temporally variable way: evidence from the US National Health and Nutrition Examination Survey, 2013-4. Journal of Obesity. 2019;2019. 10.1155/2019/7174058 PMC6791191 31662904

[36] Kowaleski-Jones L, Wen M, Fan JX (2019). Unpacking the paradox: testing for mechanisms in the food insecurity and BMI association. J Hunger Environ Nutr.

[37] Nachvak SM, Abdollahzad H, Mostafai R, Moradi S, Pasdar Y, Rezaei M (2017). Dietary diversity score and its related factors among employees of Kermanshah University of Medical Sciences. Clin Nutr Res.

[38] Nikièma V, Fogny NF, Salpéteur C, Lachat C, Kangas ST (2021). Complementary feeding practices and associated factors of dietary diversity among uncomplicated severe acute malnourished children aged 6–23 months in Burkina Faso. Matern Child Nutr.

[39] Hashimoto A, Murakami K, Kobayashi S, Suga H, Sasaki S. Associations of education with overall diet quality are explained by different food groups in middle-aged and old Japanese women.Journal of epidemiology. 2020:JE20200030. 10.2188/jea.JE20200030 PMC7940974 32418938

